# Efficacy and Safety of Direct-Acting Antivirals in Patients with Hepatitis C Infection: Nationwide Real-life Data from Türkiye

**DOI:** 10.5152/tjg.2026.25287

**Published:** 2026-04-10

**Authors:** Alper Gunduz, Nefise Çuvalcı Öztoprak, Nagehan Didem Sarı, Behice Kurtaran, Yusuf Önlen, Ebubekir Senates, Esra Zerdali, Hasan Karsen, Ayse Batırel, Rıdvan Kara Ali, Rahmet Güner, Tansu Yamazhan, Sukran Kose, Nurettin Erben, Nevin Ince, İftihar Köksal, Figen Sarıgül Yıldırım, Gulsen Yoruk, Suheyla Komur, Tayibe Bal, Sibel Kaya, Şaban Esen, Özgür Günal, Ilknur Esen Yıldız, Dilara Inan, Şener Barut, Mustafa Namıduru, Selma Tosun, Kamuran Türker, Alper Sener, Kenan Hızel, Nurcan Baykam, Fazilet Duygu, Esragül Akıncı, Güray Can, Ulku User, Hanefi Cem Gül, Ayhan Akbulut, Güven Çelebi, Mahmut Sünnetçioğlu, Oğuz Karabay, Hayat Kumbasar Karaosmanoğlu, Fatma Sırmatel, Fehmi Tabak, Hep-C Turkey Study Group

**Affiliations:** 1 Department of Infectious Diseases, Şişli Hamidiye Etfal Training and Research Hospital, İstanbul, Türkiye; 2 Department of Infectious Diseases, Antalya Training and Research Hospital, Antalya, Türkiye; 3 Department of Infectious Diseases, İstanbul Training and Research Hospital, İstanbul, Türkiye; 4 Department of Infectious Diseases and Clinical Microbiology, Çukurova University Medical Faculty, Adana, Türkiye; 5 Department of Infectious Diseases and Clinical Microbiology, Mustafa Kemal University Medical Faculty, Hatay, Türkiye; 6 Section of Gastroenterology, Medeniyet University Medical Faculty, İstanbul, Türkiye; 7 Department of Infectious Diseases, Haseki Training and Research Hospital, İstanbul, Türkiye; 8 Department of Infectious Diseases and Clinical Microbiology, Harran University Medical Faculty, Urfa, Türkiye; 9 Department of Infectious Diseases and Clinical Microbiology, Recep Tayyip Erdoğan University Medical Faculty, Rize, Türkiye; 10 Department of Infectious Diseases and Clinical Microbiology, Tekirdağ Namik Kemal University Medical Faculty, Tekirdağ, Türkiye; 11 Department of Infectious Diseases and Clinical Microbiology, Yıldırım Beyazıt University Faculty of Medicine, Ankara, Türkiye; 12 Department of Infectious Diseases and Clinical Microbiology, Ege University Medical Faculty, İzmir, Türkiye; 13 Department of Infectious Diseases, Tepecik Training and Research Hospital, İzmir, Türkiye; 14 Department of Infectious Diseases and Clinical Microbiology, Eskişehir Osman Gazi University, Eskişehir, Türkiye; 15 Department of Infectious Diseases and Clinical Microbiology, Düzce University Medical Faculty, Düzce, Türkiye; 16 Department of Infectious Diseases and Clinical Microbiology, Karadeniz Teknik University Medical Faculty, Trabzon, Türkiye; 17 Department of Infectious Diseases and Clinical Microbiology, İstanbul University-Cerrahpaşa Cerrahpaşa Medical Faculty, İstanbul, Türkiye; 18 Department of Infectious Diseases and Clinical Microbiology, Samsun 19 Mayıs University Medical Faculty, Samsun, Türkiye; 19 Department of Infectious Diseases and Clinical Microbiology, Samsun Training and Research Hospital, Samsun, Türkiye; 20 Department of Infectious Diseases and Clinical Microbiology, Akdeniz University Medical Faculty, Antalya, Türkiye; 21 Department of Infectious Diseases and Clinical Microbiology, Gaziosmanpaşa University Medical Faculty, Tokat, Türkiye; 22 Department of Infectious Diseases and Clinical Microbiology, Gaziantep University Medical Faculty, Gaziantep, Türkiye; 23 Department of Infectious Diseases, Bozyaka Training and Research Hospital, İzmir, Türkiye; 24 Department of Infectious Diseases, Bağcılar Training and Research Hospital, İstanbul, Türkiye; 25 Department of Infectious Diseases and Clinical Microbiology, Çanakkale 18 Mart University Medical Faculty, Çanakkale, Türkiye; 26 Department of Infectious Diseases and Clinical Microbiology, Gazi University Medical Faculty, Ankara, Türkiye; 27 Department of Infectious Diseases and Clinical Microbiology, Hitit University Medical Faculty, Çorum, Türkiye; 28 Department of Infectious Diseases, Ankara Dr. Abdurrahman Yurtaslan Oncology Training and Research Hospital, Ankara, Türkiye; 29 Department of Infectious Diseases, Ankara Numune Training and Research Hospital, Ankara, Türkiye; 30 Department of Gastroenterology, Bolu İzzet Baysal University Medical Faculty, Bolu, Türkiye; 31 Department of Infectious Diseases, Gülhane Training and Research Hospital, Ankara, Türkiye; 32 Department of Infectious Diseases and Clinical Microbiology, Fırat University Medical Faculty, Elazığ, Türkiye; 33 Department of Infectious Diseases and Clinical Microbiology, Bülent Ecevit University Medical Faculty, Zonguldak, Türkiye; 34 Department of Infectious Diseases and Clinical Microbiology, Yüzüncü Yıl University Medical Faculty, Van, Türkiye; 35 Department of Infectious Diseases and Clinical Microbiology, Sakarya University Medical Faculty, Hatay, Türkiye; 36 Department of Infectious Diseases, Bakırköy Dr. Sadi Konuk Training and Research Hospital, İstanbul, Türkiye; 37 Department of Infectious Diseases and Clinical Microbiology, Bolu İzzet Baysal University Medical Faculty, Bolu, Türkiye

**Keywords:** Antiviral, chronic hepatitis C, drug safety, treatment effectiveness

## Abstract

**Background/Aims:**

Chronic hepatitis C virus (HCV) infection constitutes a substantial healthcare concern in Türkiye. The clinical application of direct-acting antiviral medications (DAAs) has transformed its management. The goal is to assess the efficacy and safety of DAAs in the real-world setting in Turkish patients with chronic HCV.

**Materials and Methods:**

Thirty-seven centers from Türkiye recorded 1807 patients to the database. Patients aged >18 years were enrolled to the study. Their demographics, clinical information, DAAs used, efficacy, and safety information were evaluated. Efficacy and safety results were reported for patients with 12-week post-treatment (SVR12) data.

**Results:**

Among the patients, 919 (50.9%) were female with a mean of age 56 ± 15 years (range:18-97 years) and 238 (13%) were cirrhotic. Liver biopsy was performed in 296 patients. Mean histologic activity index score was 7.68 and fibrosis score was 2.58. Baseline mean viral load was 4.11×10^6^ copies/mL. Patients received the following treatments: Paritaprevir+Ritonavir+Ombitasvir+Dasabuvir (PrOD):706, Ledipasvir+Sofosbuvir:490, Sofosbuvir+Ribavirin:176, PrOD+Ribavirin:175, Ledipasvir+Sofosbuvir+Ribavirin:156, PrO+Ribavirin:32, and PrO:10. Response at the end of treatment was 99.2% (1454/1465) and SVR12 was 97.8% (1289/1318). The DAAs were generally well tolerated. Ten and 13 patients discontinued therapy because of drug-related and unrelated adverse side effects, respectively.

**Conclusion:**

This real-world study demonstrated that DAA treatment for HCV is both safe and highly effective. In two-thirds of the patients, the hepatic inflammation is moderate to severe, and fibrosis is moderate to advanced in half of them. Patients’ characteristics suggest that HCV infection is often not diagnosed or treated until patients present with moderate-to-severe stage, indicating that diagnostic and therapeutic approaches should be used more effectively.

**Main Points**

- In this multicenter real-world cohort from Türkiye, DAAs achieved high efficacy, with an SVR12 rate of 97.8%.
- PrOD and sofosbuvir-based regimens demonstrated comparable effectiveness, regardless of decompensation or previous treatment experience.
- DAA therapy was safe and well tolerated, with low discontinuation rates and no treatment-related mortality.

## Introduction

The global health issue of hepatitis C virus (HCV) infection is substantial, with an estimated worldwide prevalence of 71.1 million.^1^ Severe complications, such as chronic hepatitis C (CHC), liver cirrhosis, hepatic failure, and hepatocellular carcinoma (HCC) are faced by a significant number of patients with chronic HCV infections.^2^^-^^6^ These complications result in an estimated 399 000 deaths annually.^1^ Although interferon-based treatment regimens provided therapy to a certain extent,^7^ their effectiveness were limited and are associated with serious side effects. Furthermore, these agents are not used and may even be contraindicated in patients with severe disease (e.g. decompensated cirrhosis) who need urgent treatment.

The clinical use of direct-acting antivirals (DAAs) has transformed the management of HCV infection. Direct-acting antivirals appear to eradicate the virus from the blood (sustained virological response) with significantly higher frequency. In addition, these agents appear to cause much less serious adverse effects and are safe in patients with severe disease.

When patients with HCV infection achieve virologic response, the infection is eradicated, quality of life improves, and the risk of complications such as cirrhosis and HCC is reduced.^2^^,^^3^^,^^8^ Chronic HCV infection is a significant healthcare challenge in Türkiye. Anti-HCV seropositivity is 1%, and the predominant genotype is genotype 1, 1b being the most prevalent subgenotype.^4^^-^^6^ Direct-acting antivirals have been commonly available in Türkiye recently. It is ranked second in the etiology of HCC after hepatitis B virus infection.^9^^-^^11^ The objective of this multicenter prospective study was to evaluate the real-world effectiveness of direct-acting antivirals in Turkish patients with chronic HCV infection. The authors aimed to determine treatment outcomes under routine clinical conditions. In addition, the authors sought to assess the safety profile of these regimens in everyday practice.

## Materials and Methods

### Study Design

This is a prospective, non-randomized, observational multicenter cohort study.

### Patients

The current study included all consecutive patients who received DAA treatment for HCV infection and were followed up from April 1, 2017, to February 28, 2018. Patients who were previously treated (treatment-experienced) and untreated (treatment-naïve) were both included.

### Methods

**Figure 1.**
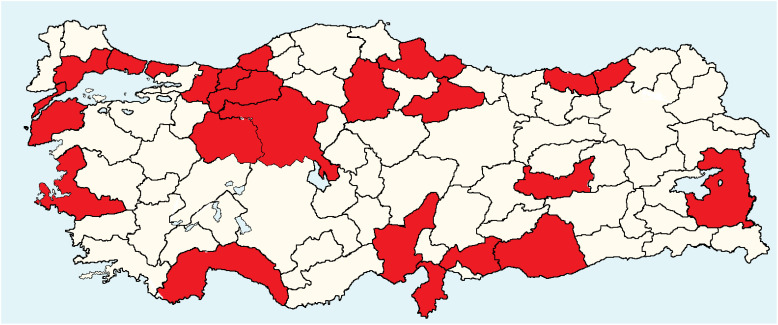
Cities of the centers included in the study.

Turkish Viral HepatitisSociety (VHSD) and Infectious Diseases and Clinical Microbiology Specialty Society (EKMUD) developed an online database to collect information on patients with Chronic Hepatitis C (CHC) undergoing DAA treatment in Türkiye. Thirty-seven centers from Türkiye recorded 1807 patients to the database. The centers were selected from multiple regions to represent the country (Figure 1). Patients aged 18 years and older with chronic hepatitis C receiving direct-acting antivirals were enrolled in this noninterventional observational trial. All patients underwent detailed history-taking (age, gender, diabetes mellitus, and other systemic comorbidities) and a clinical examination.

The following laboratory investigations were performed at baseline (within the previous 3 months), at the end of therapy (EoT) and 12 weeks after the end of therapy (sustained virologic response, SVR12): HCV RNA PCR quantitation, complete blood count, prothrombin time, serum creatinine, serum albumin, total serum bilirubin, alanine aminotransferase, alpha-fetoprotein, alkaline phosphatase, and gamma-glutamyl transferase. HCV genotype was performed at baseline for treatment-naïve patients.

This assay quantifies HCV RNA using the in vitro reverse transcription-polymerase chain reaction (PCR) method, with a sensitivity of 12 IU/mL for 0.5 mL and 30 IU/mL for 0.2 mL sample volume, with a detection range of 12 IU/mL (log 1.08 IU/mL) to 100 million IU/mL (log 8.0 IU/mL). Genotyping was completed with conventional oligonucleotide-specific primers using PCR.

A baseline abdominal ultrasound performed within the previous three months was used to assess the presence of cirrhosis and associated consequences, including atrophic liver, coarse echotexture, irregular surface, dilated portal vein, ascites, and splenomegaly. Liver biopsies were evaluated for grading and staging using Knodell’s modified method.^12^

Decompensated cirrhosis was defined as the progression of cirrhosis beyond the compensatory capacity of liver function, with symptoms such as portal hypertension, ascites, hepatic encephalopathy, or upper gastrointestinal bleeding.^13^

### Efficacy and Safety Assessment

Response to therapy was evaluated at the end of therapy (EoT) and 12 weeks post-treatment completion (SVR12) using HCV RNA PCR quantitation. Several centers investigated HCV RNA quantification at week 4 of treatment. Patients underwent regular follow-up for adverse events or abnormal findings identified during physical examinations and clinical laboratory tests. They were evaluated every 4 weeks until the end of treatment, and 12 weeks post-treatment.

### Ethical Considerations

The study was approved by the Ethics Committee of Cerrahpaşa Medical School (Date: March 07, 2017, Approval No: 59491012-604.01.02), and the study was recorded on www.clinicaltrials.gov (NCT03145844). This study was performed in compliance with the Declaration of Helsinki (2013) and the International Conference on Harmonization Guidelines for Good Clinical Practice (ICHG-GCP). Each participant provided informed consent, and their data files were anonymized and coded to ensure confidentiality.

### Statistical Analysis

Data were obtained using a Microsoft Excel database, subsequently coded, and analyzed with SPSS (Statistical Package for Social Sciences), version 15.0 (SPSS Inc.; Chicago, IL, USA). An intention-to-treat analysis was conducted. The descriptive analysis of data included percentages, means, or medians, with data presented as mean ± standard deviation (SD) or as number and percentages (%) as applicable. Kolmogorov–Smirnov test and Shapiro–Wilk test were used to assess whether the data were normally distributed. If normality was maintained, univariate analyses were conducted on all independent variables for numerical data utilizing two-sample *t*-tests, Wilcoxon signed-rank test, or Mann–Whitney *U *test, as applicable. If normality is violated, nonparametric tests were used. A *P*-value of less than .05 was established as the threshold for significance.

## Results

The data were assessed and confirmed to meet the assumption of normal distribution, allowing the use of appropriate parametric tests.

### Baseline (Pre-treatment) Patient Characteristics

The demographic characteristics, comorbidities, and laboratory results of the study population are outlined in Tables 1 and 2.

A total of 1807 patients were included in the study. Among them, 919 (50.9%) were female with a mean age of 56 ±15 years (range: 18-97 years). A total of 238 patients (13%) were cirrhotic, of whom 206 (86.5%) were compensated (Child-Pugh A) and 32 (13.5%) were decompensated (Child-Pugh B–C). Among the study group, 16% were diabetic. Genotypes were G1: 85.6% (G1b: 81%, G1a: 12%), G2 (3.8%), G3 (7.3%), G4 (2.9%), and G5 (0.3%). Liver biopsy was performed in 296. Mean histologic activity index score was 7.68, and fibrosis score was 2.58. Baseline mean viral load was 4.11×10^6^ copies/mL.

### Previous Treatments

**Figure 2.**
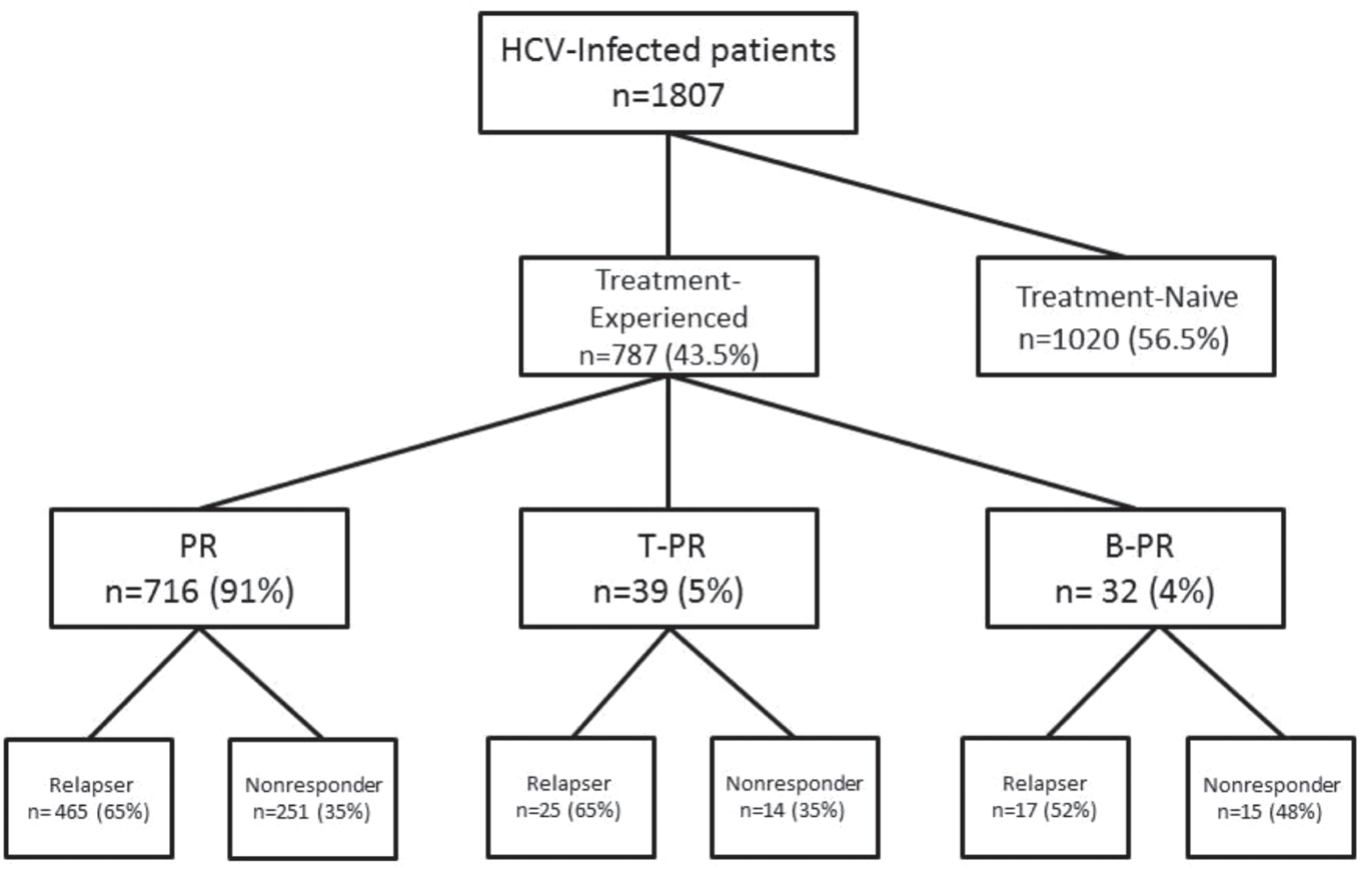
Classification of the patients according to their previous treatments. PR, pegylated interferon and ribavirin; T-PR, telaprevir and pegylated interferon and ribavirin; B-PR, boceprevir and pegylated interferon and ribavirin.

Among the study group, 1020 were treatment-naïve and 787 were treatment-experienced (61% received peginterferon + ribavirin-PR, 5% Telaprevir + PR, 4% Boceprevir + PR; and of the treatment-experienced patients, 64% were relapsers and 36% were nonresponders) (Figure 2).

**Figure 3.**
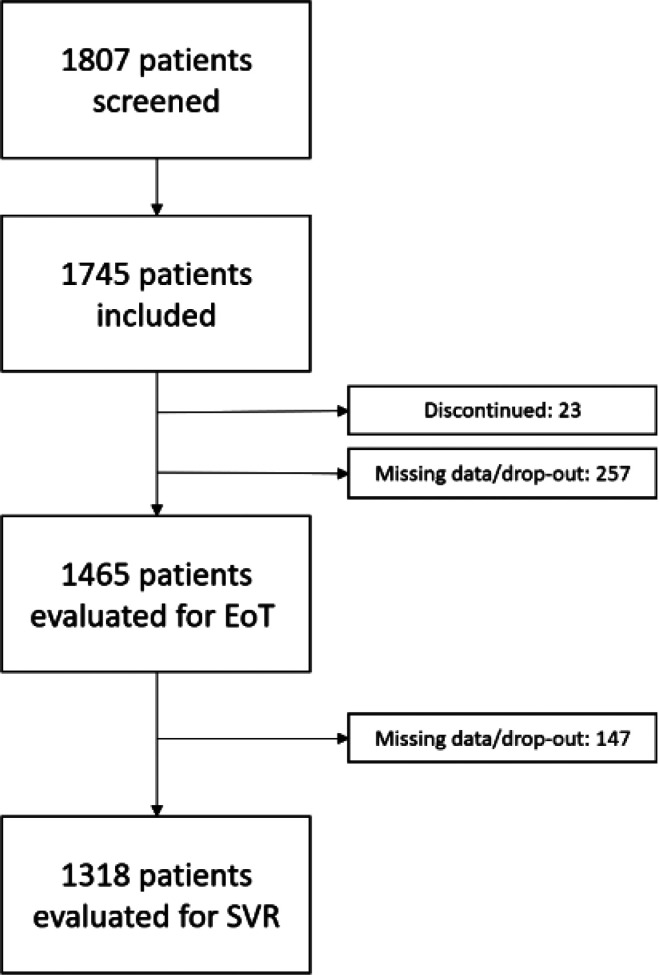
A flowchart illustrating the number of patients screened and those who received treatment.

Among the patients screened, 1745 received DAA treatment (Figure 3).

### Regimens Used

Patients were treated with Paritaprevir + Ritonavir + Ombitasvir + Dasabuvir (PrOD): 706, Ledipasvir + Sofosbuvir: 490, Sofosbuvir + Ribavirin: 176, PrOD + Ribavirin: 175, Ledipasvir + Sofosbuvir + Ribavirin: 156, PrO + Ribavirin: 32, and PrO: 10 (Table 3).

### Efficacy of Treatment

**Figure 4.**
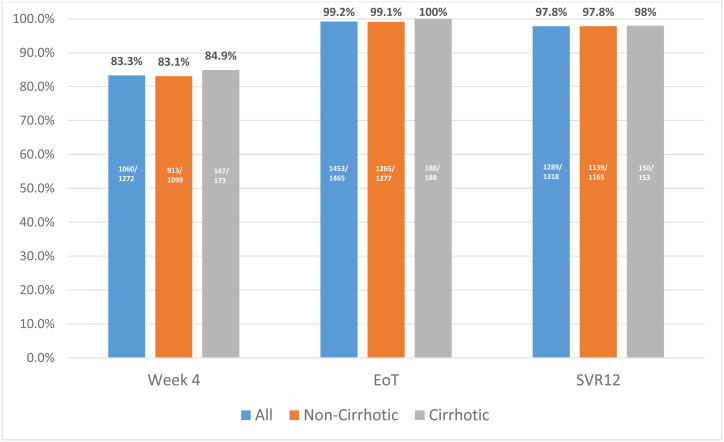
Response rates at treatment week 4, end of treatment (EoT), and 12 weeks after the completion of treatment (SVR12) in non-cirrhotic, cirrhotic, and all patients.

The week 4 virologic response was 83.3% (1,060/1,272); end of treatment response was 99.2% (1454/1465) and SVR12 was 97.8% (1289/1318) (Figure 4). Both PrOD and sofosbuvir-containing regimens provided comparable comparable efficacy. Treatment regimens and the corresponding EoT and SVR12 outcomes are presented in Table 3. The effect of treatment on the test results is displayed in Table 2. Decompensated status, regardless of treatment history (including treatment-naïve, relapse, or nonresponder), did not appear to affect the response rate (*P* > 0.5 for both).

### Safety

The DAAs were generally well tolerated. Three hundred twenty-seven patients (19.5%) described any of the following effects: asthenia (10%), pruritus (7%), nausea (3%), insomnia (3%), headache (3%), and miscellaneous (9%) (Table 4). Ten and 13 patients discontinued therapy because of drug-related and unrelated side effects, respectively. No patients among the compensated cirrhotic group experienced decompensation, and there were no mortalities during or after the treatment.

## Discussion

This real-life study primarily describes the characteristics of patients with HCV infection in the country: the disease is typically distributed evenly between sexes, predominantly with a genotype of 1b and most commonly observed in middle-aged to elderly. In two-thirds of the patients, the hepatic inflammation is moderate to severe, and fibrosis is moderate to advanced in half of them.

Patient characteristics revealed that HCV is often not diagnosed or treated until patients present with a moderate-to-severe stage, suggesting that diagnostic and therapeutic approaches should be used more effectively. However, treatment challenges in “the interferon era” should be considered. The interferon-unresponsive patient can not be treated for years, and the progression of the disease may have contributed to this group of patients with relatively advanced disease.

Real-life examples reported that ProD and sofosbuvir-containing regimens have provided an SVR of 98%. In contrast to randomized clinical trials, patients with several “exclusion criteria” are treated in real-life settings. Since DAAs have only recently become available in our country, patients who are unresponsive or contraindicated to interferon-based regimens (including those with severe comorbidities, the elderly, and decompensated cirrhotics) have accumulated. Therefore, the cohort included many difficult-to-treat patients and this high figure of SVR is meaningful for the targets of elimination of HCV.

The SVR rates of clinical trials of ProD and sofosbuvir/ledipasvir for both previously treated and untreated patients are high as in our real-life setting data.^14^^-^^16^ The safety and tolerability outcomes noted in this cohort are predominantly consistent with findings from major clinical trials.^14^^,^^16^ Adverse events were documented in 19% of patients in the cohort, in contrast to 80-90% in clinical trials, possibly due to the underreporting of recognized adverse events in clinical practice.^17^^,^^18^ The most commonly reported adverse effects in both clinical trials and real-world populations exhibited significant similarity: tiredness, itching, head pain, sleeplessness, and nausea.^14^^-^^19^

DAAs maintain a virologic response across all subgroups previously treated with interferon-based regimens.^20^ Due to severe side effects of interferon and ribavirin, elderly patients are treated with caution or remain untreated. In our cohort, 1/3 of the patients were aged 65 years and above and were treated with high efficacy and safety. Among interferon- and ribavirin-experienced patients, nonresponder patients rather than relapsers are previously regarded as difficult-to-treat patients. In the cohort, 35% of treatment-experienced patients were nonresponders; however, they responded well to DAAs.

Among cirrhotic patients, decompensated patients are considered contraindicated for interferon-based regimens. DAAs have relatively lower efficacy in decompensated cirrhotics and decompensated cirrhotics with HCC. Real-world data indicated that individuals with cirrhosis and HCC exhibit inferior SVR rates compared to cirrhotic patients without HCC.^21^^,^^22^

In a large trial, the overall SVR rates were 91% for patients without HCC and 74% for those with HCC, utilizing regimens of sofosbuvir, ledipasvir/sofosbuvir, and paritaprevir/ritonavir/ombitasvir with dasabuvir.^22^ Therapeutic regimens comprising paritaprevir, simeprevir, elbasvir/grazoprevir, glecaprevir/pibrentasvir, and sofosbuvir/velpatasvir/voxilaprevir are not appropriate for patients with decompensated cirrhosis.^23^ In the current cohort, 13.5% of the patients had decompensated cirrhosis and were treated with high efficacy and safety, considering the guideline recommendations. One of the limtations of the study was the heterogeneous nature of patients and too many treatment modalities made it difficult to analyze and compare treatment regimens. Treatment-experienced and -naïve patients, varying genotypes, and mild, moderate, and severe disease activities (compensated and decompensated patients) received several combinations of DAAs and ribavirin. A second limitation was that the analysis of efficacy was performed in patients having complete information on viral response. However, an intention-to-treat analysis results in low efficacy since those lacking virologic data are considered as unresponsive.

Third limitation of the study was that although it is a “real-life” study, comorbidities including HCC, chronic kidney disease, and liver transplantation were not reported by the participating centers. This may be explained by the fact that most participating centers were infectious disease units, where patients might have a milder disease spectrum than those typically managed in gastroenterology departments. 

Another important limitation of the current study was its inability to assess SVR in the entire study population. Although the overall cohort comprised 1,807 patients, EoT response evaluation was available for only 1,465 patients (81%), and SVR assessment was possible in just 1,318 patients (73%), primarily because of lack of follow-up and incomplete posttreatment data in the study centers of the cohort.

PrOD and sofosbuvir-containing combinations provided similar or higher rates of SVR12 in the real-world setting compared to clinical trials, with similar safety profiles.

## Figures and Tables

**Table 1.** Demographic and Baseline Characteristics of the Patients

	**HCV Patients (n = 1807)**
Male, % (n)	49.1 (888)
Age (years, mean ± SD)	56 ± 15 (range: 18-97 years)
Age >65 years, % (n)	31 (563)
HCV Gt, % (n)	
Gt1	86.5 (1547)
Gt1a	(193)
Gt1b	(1257)
Gt1-undetermined	(97)
Gt2	3.8 (68)
Gt3	7.3 (132)
Gt4	2.9 (53)
Gt5	0.3 (5)
Gt-undetermined	0.1 (2)
HCV RNA (copies/mL, mean)	4.11×10^6^
Treatment-experienced, % (n)	43.6 (787)
Cirrhosis, % (n)	13 (238)
Child-Pugh A	86.5 (206)
Child-Pugh B-C	13.5 (32)
Diabetic, % (n)	16.2 (293)
Liver biopsy, % (n)	16.4 (296)
HAI score (mean)	7.68
Fibrosis score (mean)	2.58

Gt, genotype; HAI, histologic activity index; HCV, hepatitis C virus.

**Table 2.** Laboratory Parameters at Baseline, On Treatment, and After the Treatment

	**Baseline**	**Month 1**	**EoT**	**SVR12**
ALT (U/L)	55 ± 50	22 ± 22	20 ± 19	19 ± 16
AST (U/L)	48 ± 39	24 ± 17	22 ± 12	22 ± 12
Albumin (g/dL)	5.63	4.15	4.24	4.25
PT (sec)	13.6	12.6	12.5	12.4
INR	1.78	1.08	1.06	1.07
Platelets (/mm^3^)	171 469	178 242	181 692	178 467

EoT, response rate at the end of therapy; SVR12, response rate 12 weeks after the end of therapy; ALT, alanine aminotransferase; AST, aspartate aminotransferase; PT, prothrombin time; INR, international normalized ratio.

**Table 3.** Treatment Regimens and Corresponding Virologic Response Rates

**Treatment Regimen**	**n (%)**	**EoT (%)**	**SVR12 (%)**
Paritaprevir + Ritonavir + Ombitasvir + Dasabuvir	706 (40.5)	98.9	98.1
Ledipasvir + Sofosbuvir	490 (28.1)	99.5	98.7
Sofosbuvir + Ribavirin	176 (10.1)	100	95.6
Paritaprevir + Ritonavir + Ombitasvir + Dasabuvir + Ribavirin	175 (10.0)	100	99.2
Ledipasvir + Sofosbuvir + Ribavirin	156 (8.9)	99.3	98.4
Paritaprevir + Ritonavir + Ombitasvir + Ribavirin	32 (1.8)	100	90.0
Paritaprevir + Ritonavir + Ombitasvir	10 (0.6)	85.6	60.0

EoT, response rate at the end of therapy; SVR12, response rate 12 weeks after end of therapy.

**Table 4.** Main Side Effects of the Treatment Regimens

**Adverse Event/Treatment Regimen**	**Ledipasvir + Sofosbuvir**	**Ledipasvir+Sofosbuvir+Ribavirin**	**Paritaprevir+ Ritonavir+Ombitasvir**	**Paritaprevir+Ritonavir+Ombitasvir+Dasabuvir**	**Paritaprevir + Ritonavir+ Ombitasvir + Dasabuvir + Ribavirin**	**Paritaprevir +Ritonavir+Ombitasvir+Ribavirin**	**Sofosbuvir + Ribavirin**	**Total**
All	90/466	31/153	0/10	130/674	39/164	4/30	28/160	322/1657
Fatigue	47/443	21/135	0/10	43/663	24/151	2/30	20/156	157/1588
Pruritus	21/469	7/149	0/10	68/638	8/167	1/31	8/168	113/1632
Headache	20/470	8/148	0/10	14/692	5/170	0/32	4/172	51/1694
Insomnia	10/480	8/148	0/10	21/685	8/167	0/32	7/169	54/1691
Nausea	12/478	5/151	0/10	27/679	9/166	1/31	4/172	58/1687
Others	42/448	15/141	0/10	49/657	22/153	1/31	14/162	143/1602

## Data Availability

The data that support the findings of this study are available on request from the corresponding author.
